# ‘Talk CKD Options’: improving how clinicians communicate treatment options in advanced kidney disease using a conversation analysis-based training intervention

**DOI:** 10.1186/s12882-026-05240-1

**Published:** 2026-08-11

**Authors:** Samuel Westaway, Chloe B. Shaw, Barnaby Hole, Fliss E. M. Murtagh, Fergus J. Caskey, James A. Tulsky, Ruth Parry, Rebecca K. Barnes, Lucy E. Selman

**Affiliations:** 1 Palliative and End of Life Care Research Group, Bristol Medical School, Bristol, UK; 2 Renal Research Group, Bristol Medical School, Bristol, UK; 3 Bristol NHS Foundation Trust, Bristol, UK; 4 Wolfson Palliative Care Research Centre, Hull York Medical School, University of Hull, Hull, UK; 5 Dana-Farber Cancer Institute, Harvard Medical School, Boston, USA; 6 Centre for Research in Communication and Culture, School of Social Sciences and Humanities, Loughborough University, Loughborough, UK; 7 Nuffield Department of Primary Care Health Sciences, University of Oxford, Oxford, UK; 8 Renal and General Internal Medicine Higher Specialty Trainee, Bristol NHS Foundation Trust, NIHR Academic Clinical Fellow University of Bristol, Bristol, UK

**Keywords:** Communication, Conservative kidney management, Dialysis, Elderly, Quality of life

## Abstract

**Background:**

Most older people with kidney failure and multiple co-morbidities must decide between dialysis or conservative kidney management. Dialysis is often presented as the default, despite providing uncertain survival benefits in this population. The Optimising Staff-Patient Communication in Advanced Renal Disease (OSCAR) study used conversation analysis of real consultations to develop a communication training intervention to support the provision of person-centred shared decision-making: ‘Talk CKD Options’. This pilot study aimed to: (i) deliver the intervention and (ii) assess its feasibility and acceptability.

**Methods:**

Talk CKD Options comprised a 3-hour online, interactive session, plus two pre-recorded pre-training presentations. Pre-training surveys explored experience, previous training, and self-reported knowledge, skill and confidence. Post-training surveys assessed self-reported outcomes and gauged opinions on the training. Semi-structured interviews were conducted ≥ 2 weeks post-training. Interview data were analysed using thematic analysis.

**Results:**

Twenty-seven clinicians from 20 English renal centres attended: 9 doctors, 17 nurses, 1 psychologist. 74% had no previous training in communicating treatment options. Post-training surveys demonstrated improved self-reported knowledge (*p* < 0.001), skill (*p* = 0.002) and confidence (*p* = 0.001) in communicating about treatment options; all reported it would influence their practice. In interviews the value of multidisciplinary team training and breakout group-work were highlighted as strengths. Participants desired a summary handout and more breakout group-work.

**Conclusions:**

Talk CKD Options demonstrates novel use of conversation analysis-based communication training in advanced kidney disease. That only one quarter of our motivated participants had formal training in this area, suggests a training need. It was highly rated, improving self-reported knowledge, skill and confidence. Talk CKD Options provides renal clinicians with tools that aim to support the provision of person-centred shared decision-making.

**Supplementary Information:**

The online version contains supplementary material available at https://doi.org/10.1186/s12882-026-05240-1.

## Background

Transplantation is rarely an option for older people with kidney failure [1], who instead receive treatment with conservative kidney management (CKM) or dialysis. CKM focuses predominantly on promoting quality of life, but does not include kidney replacement therapy [2]. Observational data suggests survival advantages are comparable in patients with frailty, multimorbidity, and/or aged over 80, whether treated with CKM or dialysis [3–6]. Increased treatment burden with dialysis is also well described in the older population [3, 6, 7]. Nevertheless, research highlights inconsistency and bias in communication about dialysis versus CKM [8–10]. Inter-centre variation in CKM practice and uptake has been reported [11], with variable communication of treatment options proposed as one underlying factor. Guidelines advocate offering treatment choice and ensuring decisions are made jointly, taking into account a person’s preferences [12]. A 2025 narrative review of patient-physician communication in advanced kidney disease, emphasised the “critical need for improved communication skills training” [13]. A 2023 systematic review on factors that influence the selection of CKM cited the limited clinical guidance and training on CKM as a reason dialysis may be offered to most patients by default [14]. 

The Optimising Staff-Patient Communication in Advanced Renal Disease (OSCAR) study was a 5-year multi-phase study which aimed to enable the consistent provision of person-centred treatment decision-making support and reduce unwarranted treatment variation through the development of an evidence-based communication training intervention for renal clinicians: ‘Talk CKD Options’. OSCAR followed the Medical Research Council guidelines for complex intervention development [15], and the ‘person-based’ approach to the development of behaviour change interventions [16]. The initial phase involved observing standard clinical practice (ethnographic observation) and undertaking clinician interviews at four UK renal units, followed by video-recordings of outpatient consultations, plus quantitative and qualitative data collection from clinicians, patients and caregivers in the recordings. Differing communication practices in the video-recorded consultations were identified using conversation analysis; [17] a systematic evidence-based method that enables the interactional consequences of differing communication practices for the same action to be identified, and for linkages with contexts and outcomes to be subsequently investigated.

OSCAR showed that a cluster of communication practices tended to present CKM as less favourable or suitable than dialysis; this could influence people’s ability to apply what is important to them to the decision. We found a tendency for clinicians to minimise dialysis risks [18], and for patient information documents to present unbalanced explanations of dialysis and CKM, with the latter framed as a ‘non-treatment’ linked to death [19]. In the video-recordings, two main communication practices when presenting treatment options were identified: (1) CKM and dialysis being presented as the main treatment options; (2) CKM being presented as a subordinate option to dialysis [20]. 

OSCAR study findings were integrated to develop a prototype intervention with stakeholders, Talk CKD Options, which was refined via think-aloud interviews with renal clinicians and feedback from patient representatives and stakeholders. The three key takeaway messages underlying the training in Talk CKD Options are: (1) Know the evidence, (2) Present treatment options equitably, (3) Allow people’s values to drive the decision; full learning objectives are available as an additional file (Additional file 1).

This pilot study aimed to: (i) deliver the Talk CKD Options training intervention and (ii) test its feasibility and acceptability among renal clinicians.

## Methods

Talk CKD Options comprised a 3-hour online, live and interactive session delivered by facilitators with content expertise (authors LES, CS, BH). It was supplemented by two pre-recorded presentations by facilitators with relevant clinical and research expertise (FEMM, BH) to be watched in advance. Our mixed-methods evaluation comprised online pre- and post-training surveys, followed by a single semi-structured interview conducted ≥ 2 weeks post-training.

### Participants and recruitment

Recruitment aimed for a sample size of 15–30 via emails to national special interest groups and additionally through stakeholder and collaborator networks. The minimum size of 15 was based on literature suggesting this would be sufficient to attain the informational power required [21], given the anticipated diversity in learning preferences and needs. The maximum size of 30 was set to enable online-training facilitation. As a pilot study, focused on intervention acceptability and feasibility, a formal sample size calculation for testing intervention effectiveness was not performed. Purposive sampling was used aiming for diversity in participant role (medical consultant/registrar/specialist nurse) and represented centres. Clinicians from across the multidisciplinary team (MDT) were eligible if they provided care to people with an eGFR < 20 ml/min/1.73m^2^ and discussed kidney failure treatment decision-making.

A £50 Love2Shop voucher was offered to clinicians who completed all evaluation elements. All participants provided written informed consent prior to pilot attendance. This research was conducted in accordance with the Declaration of Helsinki and ethical approval for this phase of the OSCAR study was granted by the University of Bristol Faculty of Health Sciences Research Ethics Committee (Ref: 17890).

### Data collection

Surveys were informed by and adapted from our prior research [22, 23]. The pre-training survey explored experience and previous training about communicating with patients regarding treatment options for advanced kidney disease. Participants also self-reported knowledge, skill and confidence in this topic. The post-training survey re-assessed those self-reported metrics, and collected quantitative and qualitative feedback on training format, structure and delivery. Surveys are provided as additional files (Additional files 2 and 3).

Qualitative semi-structured interviews followed a topic guide (Additional file 4) developed in conjunction with a wider project advisory group including patient and public involvement representatives and clinicians, educators and researchers with relevant content-expertise (Acknowledgements). Interviews were planned for ≥ 2 weeks post-training on Microsoft Teams one-to-one with a male researcher (SW) who had not delivered the training. This delay allowed implementation of any learning into practice and assessment of knowledge retention. The researcher (SW) had communicated with participants prior to interview, when providing pilot information and collecting written informed consent in his stated role as a trainee renal doctor and researcher with the University of Bristol. Microsoft Teams’ auto-transcription was used; a researcher (SW) checked transcript accuracy, anonymising transcripts post-interview, whilst retaining participants’ roles: D-*doctor*, N-*nurse* or P-*psychologist*.

### Data analysis

StataMP 18 was used for statistical analyses, using a non-parametric test (Wilcoxon Signed-Rank Test) to analyse pre- and post-training Likert scale data as an entire cohort and for doctors and nurses separately.

Semantic thematic analysis of qualitative data from interviews and free-text post-training survey responses used a combination of deductive coding, with pre-specified themes relevant to the pilot evaluation objectives, and inductive coding of participant responses. Pre-specified themes to be explored were: (i) Impact on practice, (ii) Potential impact on patient outcomes, (iii) Acceptability and feasibility of each component of the training, (iv) Barriers to implementation, limitations of training and suggestions for improvements. Within acceptability and feasibility, five pre-specified sub-themes were: (i) Amount of content, (ii) Breakout groups, (iii) Length, (iv) MDT learning, and (v) Online delivery. A team approach was used, with three researchers (SW, CS, LES) independently coding the first three transcripts then meeting to refine a coding framework. The primary researcher (SW) then applied the framework to the qualitative data from each participant’s post-training survey and semi-structured interview within Nvivo v.14. The Consolidated criteria for reporting qualitative research (COREQ) checklist was applied (Additional file 5) [24]. 

## Results

### Participant characteristics

Twenty-eight clinicians consented to participation, with recruitment completed in 32 days. Twenty English renal centres were represented across varied geographical regions (South West: 4, North West: 4, South East: 3, North East: 3, Midlands: 3, London: 2 and East of England: 1), serving populations in which the proportion of individuals of White ethnicity ranged from 46% to 96% [25]. 

Twenty-seven (96%) attended and completed a pre-training survey: 8 consultant nephrologists, 1 registrar, 17 nurses and 1 clinical psychologist (Table 1). In the UK, consultants are fully qualified hospital-based specialists who practice independently, whilst registrars have chosen their specialty but are still in training. Attendees had a median of 5 years’ experience with a specific role in CKD care (IQR 3–10); 74% had received no formal training in communicating with patients about treatment options. Most attendees were white (81%) and women (81%).

**Table 1:** Participant demographics

	Number	Percentage
***Clinician Type***		
Nurse	17	63%
Consultant	8	30%
Registrar	1	4%
Psychologist	1	4%
***Years in CKD care role***		
Less than 2	6	22%
2 to 5	8	30%
5 to 10	7	26%
More than 10	6	22%
***Prior training in communicating about treatment options for advanced kidney disease***		
Yes	7	26%
No	20	74%
***Gender***		
Woman	22	81%
Man	5	19%
Non-binary	0	0%
Prefer to self-describe	0	0%
***Ethnicity***		
White	22	81%
Mixed	0	0%
Asian	3	11%
Black	0	0%
Other	2	7%
***Total***	27	100%

Twenty-five (93%) attendees submitted a post-training survey and 26 (96%) completed a semi-structured interview post-training (median 27 days post-training, IQR 15–32, range 14–89 with a high upper range limit due to unplanned post-training leave for one participant). Median interview length was 13 min (IQR 11–14). Post-training quantitative survey results, including comparative results with pre-training data, include only those 25 who completed both pre- and post-training surveys. Qualitative data is included from all 26 attendees who attended a semi-structured interview, as well as from qualitative responses in survey questions.

### Findings

Both the qualitative and quantitative results are reported with reference to the prespecified evaluation topics. A qualitative analysis coding summary table is provided as an additional file (Additional file 6).

### Impact on practice and self-reported outcomes

**Fig. 1:**
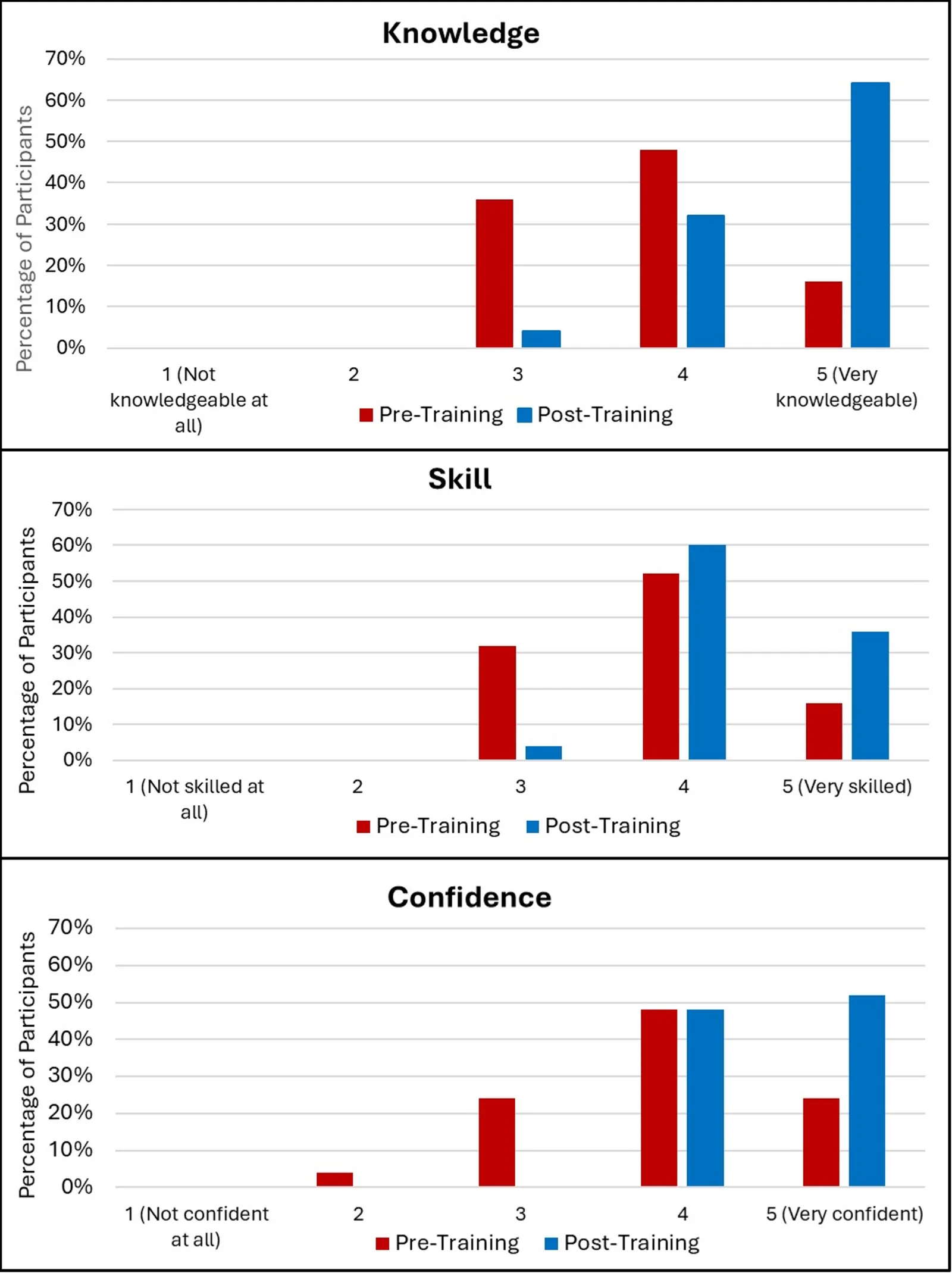
Self-reported levels of Knowledge, Skill and Confidence in communicating with patients about treatment options for advanced kidney disease pre- and post-training

Pre-training, the most common participant response when asked to rate their knowledge, skill and confidence in communicating with patients about treatment options for advanced kidney disease was 4 out of 5 in all three categories (Table 2). Comparing pre- and post-training surveys demonstrated significant improvements in self-reported knowledge (*p* < 0.001), skill (*p* = 0.002) and confidence (*p* = 0.001, Table 2; Fig. 1). Improvements were still observed when stratified by clinical role, with statistically significant improvements observed for all self-reported categories for the 16 nurses, but only in improved confidence for the smaller sample of 8 doctors. No statistical testing was performed on the single psychologist’s ratings.

**Fig. 2:**
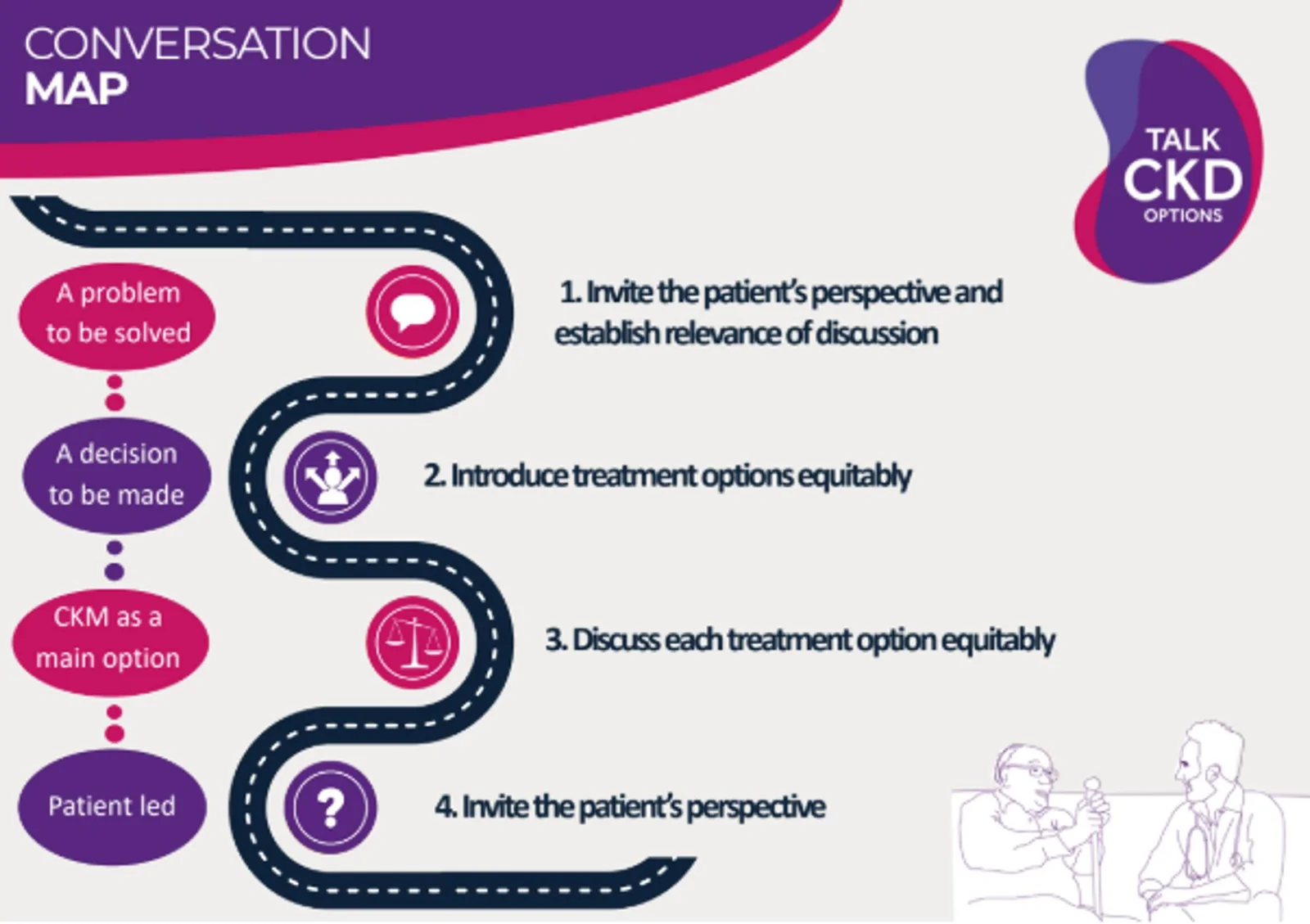
Conversation map – communication tool presented in Talk CKD Options

**Table 2:** Summary of self-reported knowledge, skill and confidence levels pre- and post-training

**My level of knowledge in communicating with patients a** **bout treatment options for advanced kidney disease**						
	Pre-Training		Post-Training			
Knowledge Level	*N*	%	*N*	%	*p*-value*	Participant Quote
1 (Not knowledgeable at all)	0	0%	0	0%	*p <* 0.001	In relation to training content: *“Very clear*,* evidence-based statistics that* [are] *transferable to be used with patient education.”* N10
2	0	0%	0	0%		
3	9	36%	1	4%		
4	12	48%	8	32%		
5 (Very knowledgeable)	4	16%	16	64%		
**My level of skill in communicating with patients ** **ab** **out treatment options for advanced kidney disease**						
	**Pre-Training**		**Post-Training**			
**Skill Level**	**N**	**%**	**N**	**%**	***p-value***	**Participant Quote**
1 (Not skilled at all)	0	0%	0	0%	*p =* 0.002	*“I really like having the conversation map as it is very useful to have a way of structuring those difficult conversations.”* N6
2	0	0%	0	0%		
3	8	32%	1	4%		
4	13	52%	15	60%		
5 (Very skilled)	4	16%	9	36%		
**My level of confidence in communicating with patients ** **ab** **out treatment options for advanced kidney disease**						
	**Pre-Training**		**Post-Training**			
**Confidence Level**	**N**	**%**	**N**	**%**	***p-value***	**Participant Quote**
1 (Not confident at all)	0	0%	0	0%	*p =* 0.001	*“I very much feel more confident.”* N14
2	1	4%	0	0%		
3	6	24%	0	0%		
4	12	48%	12	48%		
5 (Very confident)	6	24%	13	52%		

*p-values from Wilcoxon Signed-Rank Test to analyse pre- and post-training Likert scale data

When reflecting on what they felt the training’s takeaway messages were, four recurring themes were identified: the importance of discussing treatment options equitably, adopting a person-centred approach, attending to language, and knowing the evidence. This correlated well with the training’s three key takeaway messages: (1) Know the evidence, (2) Present treatment options equitably, (3) Allow people’s values to drive the decision. D7 summarised their learning as *“presenting conservative kidney management as an equal option… then sort of coming to a shared decision driven by values and preferences and what’s important to the patient.”* Regarding the importance of language, N2 reported the training *“really made me think about how we talk to patients and how what we actually say and the words we use can influence their decisions on things”.*

In the survey, all reported the training would influence their practice, with 68% saying “to a great extent”. A positive impact on changing practice was consistently reinforced during interviews. N12 explained, *“what is important for the patient? What is their priority? That has become more of an emphasis for me*,* definitely.”* Participants described directly hearing from patients, through patient interviews and via the real consultation clips, as being powerful, with D1 saying they were *“most inspired by watching the patient videos”.*

Another impact on practice related to sharing knowledge with others, with 8 clinicians describing how they fed-back their learning within their teams. N4 said *“I’ve been banging on about it to everybody that will listen”* and N14 described how they *“did like a mini-session about this… the training and the content briefly.”*

Regarding which staff would benefit most, the majority felt the training would be beneficial across the MDT and seniority levels. At interview, when exploring potential training impact, D5 believed *“it is really crucial for kind of everyone within the renal community to be receiving training like this”.* It was felt to be particularly beneficial to new starters and registrars, with N15 stating *“it would have been something so*,* so valuable when I was first starting”.*

### Potential impact on patient outcomes

In interviews, there was consensus that the training could impact outcomes including treatment choice, although 3 responders highlighted measuring training impact could be difficult. When asked if they thought the training had the potential to benefit patient outcomes, P1 suggested *“In terms of people’s own values base being promoted and sort of respected then absolutely you could see that being the case. I could also see the case of more people choosing conservative management as an option based on this training.”*

### Acceptability and feasibility of each component of the training

When surveyed, approximately 90% agreed that all learning objectives had been met to a reasonable or great extent (Table 3). 96% were likely to recommend the training to a colleague (Table 4), including D6 who had *“told everyone about it… it was really good.”*

**Table 3:** Were the learning objectives of the training met?

**Summarise the existing observational evidence about dialysis outcomes in the elderly and/or multimorbid population**		
Not at all	0	0%
To a very minimal extent	0	0%
Somewhat	2	8%
To a reasonable extent	7	28%
To a great extent	16	64%
**Understand how older people make the decision about treatment options in advanced kidney disease**		
Not at all	0	0%
To a very minimal extent	0	0%
Somewhat	3	12%
To a reasonable extent	7	28%
To a great extent	15	60%
**Identify how the way we work and the environment we work in can impede our ability to support people**		
Not at all	0	0%
To a very minimal extent	0	0%
Somewhat	1	4%
To a reasonable extent	8	32%
To a great extent	16	64%
**Develop a conversation map and toolkit to provide person-centred support for treatment decision making in advanced kidney disease**		
Not at all	0	0%
To a very minimal extent	1	4%
Somewhat	1	4%
To a reasonable extent	7	28%
To a great extent	16	64%

**Table 4:** Overall feedback

**How likely would you be to recommend the training to a colleague?**		
Very Unlikely	0	0%
Unlikely	0	0%
Neutral	1	4%
Likely	5	20%
Very Likely	19	76%
**To what extent do you think the training will influence your practice?**		
Not at all	0	0%
To a very minimal extent	1	4%
Somewhat	3	12%
To a reasonable extent	4	16%
To a great extent	17	68%

#### Amount of content

Most believed the training included the right amount of content and when surveyed, all deemed the consultation examples to be a good amount (Table 5). However, at interview, 6 nurses reported feeling there was too much information, including N9 who felt *“too much information was delivered*,* and it was hard to retain all the information”.*

**Table 5:** Acceptability and feasibility of each component of the training

**To what extent do you think the overall mode of the training (being online, live and interactive) worked well?**		
Not at all	0	0%
To a very minimal extent	0	0%
Somewhat	1	4%
To a reasonable extent	12	48%
To a great extent	12	48%
**If the training was online**,** but you could access it in your own time (rather than being live and interactive)**,** to what extent do you think that would work well?**		
Not at all	1	4%
To a very minimal extent	2	8%
Somewhat	7	28%
To a reasonable extent	11	44%
To a great extent	4	16%
**To what extent do you think training as a multidisciplinary team worked well? (as opposed to only training with other doctors/nurses etc.)**		
Not at all	0	0%
To a very minimal extent	0	0%
Somewhat	3	12%
To a reasonable extent	4	16%
To a great extent	18	72%
**The overall length of the live training session was…**		
Far too short	0	0%
Slightly short	3	12%
A good length	19	76%
Slightly long	2	8%
Far too long	1	4%
**The number of consultation video examples used was…**		
Far too few	0	0%
Slightly too few	0	0%
A good amount	25	100%
Slightly too many	0	0%
Far too many	0	0%
**How would you rate the breakout group sessions?**		
Very Poor	0	0%
Poor	0	0%
Fair	3	12%
Good	7	28%
Very Good	15	60%

#### Breakout groups

Good facilitation of the breakout groups was consistently reported during interviews and the breakout group-work was rated “Good” or “Very good” by 88% (Table 5). One of the five nurses who expressed a reluctance to contribute in big groups when interviewed, reported feeling their group was *“very dominated by the doctors”* (N9). N16 stated that they *“liked that we had the breakout rooms twice. I struggle to speak in a large group of people”.*

#### Length

When surveyed, 76% deemed the training session to be a “good length”, with a minority (12%) suggesting the training was slightly short (Table 5). At interview, P1 explained *“there was so much to discuss… it was really interesting… we wanted a bit more”.*

#### MDT learning

Positive attitudes towards and experiences of MDT learning were clear, with 88% reporting training as an MDT worked well to a “reasonable” or “great extent” (Table 5). Despite this, 4 clinicians discussed the impact of perceived hierarchy at interview. When asked “How did you find training as an MDT?”, N4 stated that *“it can be a little bit daunting as a nurse sometimes*,* when you’ve got some really - kind of - senior people*,* as there [were] nephrologists and such”.*

#### Online delivery

96% believed the online, live and interactive format worked well to a “reasonable” or “great extent”. The majority were positive about online delivery; a key factor here was providing access to a wider cohort. D3 described that *“it’s nice to have a face to face but actually one of the advantages of doing it online is there were people from different units there. And so you get a wider perspective.”*

### Barriers, limitations and suggestions for improvements

#### Barriers to implementation

In the qualitative data, barriers to implementing learning from the training were identified in relation to patient factors and system factors. The most frequently referred-to patient-related factor was the impact of pre-conceived ideas patients and families had about treatments, described by 9 responders. When asked about barriers to training implementation, N2 outlined, *“barriers… probably come from people*,* stuff that people already know before*,* preconceived ideas about things… they kind of already come with a preconceived idea that they’ve got to have dialysis”.* Linked to this, participants described how conflicts between clinicians and families and cultural barriers could act as barriers to implementing the learning. Regarding conflicts with families, N3 outlined *“we have families saying no*,* you have to have dialysis. That can be a barrier*,* a very difficult one”.* Cultural barriers were raised by 3 clinicians; for example, N8 described *“for some cultures it might be that conservative management does feel like giving up”*.

Within system factors, lack of time was most consistently reported, featuring in 9 interviews. D3 explained, *“the main thing is just having the time to have those slightly richer*,* slower conversations”.* The variation in CKM services offered at different units was the other most notable system-related barrier, reported by five participants. D9 outlined: *“variability in services is a barrier to doing it well and you know you might have other places you’ve maybe got supportive care nurses”.*

#### Limitations of training

The most common limitation identified was retaining knowledge of the conversation map (Fig. 2); eight participants reported at interview that they failed to recall it. N10 stated, *“I definitely remember thinking that’s a really good tool and will be really good to use it. But actually funnily enough I haven’t then kind of thought about that element again.”*

Two participants highlighted that the training didn’t cover cultural differences in communication practices with N9 feeling *“The scenarios did not reflect a diverse population*… *working with a demography of different backgrounds*,* ethnic backgrounds*,* different belief systems and things. I think none of that was kind of mentioned.”*

#### Suggestions for improvements

When asked about improvements, approximately half requested supplemental materials, expressing a desire for a post-session handout and training slides access. D7 suggested *“Give a resource of example statements or questions which clinicians can use for each stage of the conversation map”.*

The minority who suggested lengthening it proposed creating two sessions, more time on communication tools and breakout group-work. N2 felt it was *“a little bit too much to try and cover in three hours. So maybe if they’d have been like maybe two sessions or even like two 2-hour sessions or something.”* Regarding breakout group-work, N13 said *“…where we were separated by groups felt a bit short as I think due to the diverse backgrounds*,* more sharing of ideas could have been achieved.”* Three clinicians specifically suggested role-play opportunities would enhance the training, with D1 stating *“consider a FU [follow up] session to role-play some of the skills learnt.”*

## Discussion

Talk CKD Options was highly rated, improving self-reported knowledge, skill and confidence in communicating treatment options and influencing the practice of all attendees. The core training elements – online, live, interactive delivery incorporating breakout groups in an MDT format – were very well received. Patients’ and families’ preconceptions around the suitability and effectiveness of dialysis and CKM were frequently identified by participants as a barrier to implementing learning from the training. A better trained workforce might be able to address preconceptions at an earlier stage, through consistent and equitable presentation of kidney failure treatments across the disease course. Given three quarters had never received formal training in this area, this intervention has the potential to provide for unmet training needs and improve practice.

Previous attempts to improve communication and shared decision-making in this context are limited. A 2016 systematic review of communication skills training for non-specialist palliative care providers identified two renal-specific interventions: [26, 27] REACT [28] and NephroTalk [29]. REACT was deemed acceptable and feasible in a UK pilot study, with non-significant confidence benefits, whilst in the US NephroTalk improved preparedness in delivering bad news, expressing empathy and discussing dialysis. A further pilot study has since successfully used a the ‘Best case/worst case’ tool to improve shared decision-making about dialysis for the over-70s, albeit in a small sample of 16 nephrologists [30]. Lastly, a Taiwanese study demonstrated improvements in short-term ‘truth-telling confidence’ but not in shared decision-making ability, using an online training programme [31]. 

Unlike Talk CKD Options, these previous interventions were not grounded in direct observational evidence about the structure and functioning of renal clinicians’ actual communication. The Talk CKD Options format is novel within the field, using video-recordings of real consultations within the training, and conversation analysis to identify relevant practices for shared decision-making. Conversation analysis-based materials to support communication training have been used elsewhere, including: improving psychiatrists’ communication with patients with psychosis [32], addressing unmet patient concerns in primary-care [33], supporting communication with patients with dementia [34] and optimising prudent prescribing [35]. It therefore represents a previously untapped methodology to provide evidence-based communication training in advanced kidney disease treatment decision-making; this pilot study demonstrates it is a feasible and acceptable approach among renal clinicians, with potential impact on practice and patient outcomes, warranting further research.

Study findings provide valuable suggestions for improvements, including producing a handout and providing access to additional consultation examples not covered. This may help address the problem of knowledge retention identified, specifically of the conversation map. A handout could also provide a potential reference for use in consultations. The suggestions to lengthen the training into two sessions would facilitate an opportunity for more breakout group-work, providing a chance for discussion regarding challenging scenarios highlighted by participants as not covered in the pilot, such as differing cultural beliefs and conflict with families.

When training a range of MDT members, awareness of perceived power dynamics is vital. Although MDT learning was overwhelmingly positively received, perceived hierarchy and reluctance from some to contribute in big groups might have limited the ability of some’ participants to engage – a phenomenon that appeared to be especially experienced by nurses. These reflections will be integrated into future facilitator guidance to create a ‘flattened hierarchy’, where all MDT members feel their perspectives are welcomed [36]. Existing research suggests a psychologically safe learning environment where all participants feel empowered to contribute can be created through the explicit downplaying of power differentials [37]. Future training introductions will place greater emphasis on this point.

### Limitations

In this pilot study, our focus has been on acceptability, feasibility and training delivery, including potential impact on practice and suggestions for improvements to inform future refinement and evaluation. The improvements in knowledge, skill and confidence described are self-reported and assess participants’ training reactions, a basic level of training assessment. There is scope to expand the remit to include more advanced evaluation metrics, assessing impact via: serial assessments, behaviour in clinical scenarios, patient-level outcomes such as treatment choice and patient-reported outcomes including perceptions of consultations [38]. The transfer of clinician-reported training reactions to patient-level outcomes such as treatment choice is complex; [39] identified barriers to implementation such as pre-conceived ideas about treatments show patient-level outcomes are influenced by multiple factors, not solely clinician communication. The inclusion of patient-reported perceptions of consultations, for example via the Shared Decision-Making Questionnaire (SDM-Q-9) we have previously used in this context [20, 40], will be vital to understanding training efficacy in future evaluations.

Specialist nurses play a key role in informing patients’ treatment decision-making, for example in how nurses describe and frame treatment options, while doctors commonly lead treatment decision-making discussions. Our study sample included 17 nurses and 9 doctors, of whom only one was a registrar. Future evaluation should ensure the perspectives of doctors, particularly trainees, are fully explored.

Attendees were contacted through relevant networks and likely to be interested in this topic. This makes them less likely to be representative of a wider cohort, also suggested by high baseline levels of self-reported ability. This group is likely to be accepting of CKM as a valid alternative to dialysis; evidence exists that some clinicians are unconvinced of this [14, 41]. Most participants were white and most were women. Future training evaluations should include more sceptical clinicians, from a range of demographic backgrounds.

In conclusion, Talk CKD Options was successfully piloted as a novel communication training intervention for clinicians supporting older people with treatment decision-making in advanced kidney disease. For the first time in renal medicine, we employed conversation analysis to identify communication practices that promoted the presentation of equitable options and integrated these into training. Talk CKD Options meets the critical need for improved communication skills training in this area; access to it should now be widened to a more heterogenous cohort, representing renal clinician diversity. Our results suggest more detailed evaluation of the intervention’s effectiveness is now warranted, informed by this study’s findings.

## Supplementary Information

Below is the link to the electronic supplementary material.

Supplementary Material 1: Additional file 1: Learning objectives.

Supplementary Material 2: Additional file 2: Pre-training online survey.

Supplementary Material 3: Additional file 3: Post-training online survey.

Supplementary Material 4: Additional file 4: Clinician interview topic guide: Pilot intervention.

Supplementary Material 5: Additional file 5: Consolidated criteria for reporting qualitative research (COREQ) checklist.

Supplementary Material 6: Additional file 6: Coding framework for qualitative data analysis.

## Data Availability

The data used and analysed during the current study are available from the corresponding author upon reasonable request.
