# Supplementary material for: ‘Talk CKD Options’: improving how clinicians communicate treatment options in advanced kidney disease using a conversation analysis-based training intervention
Source: BMC Nephrol. 2026 Aug 11;27:656. doi: 10.1186/s12882-026-05240-1 (PMC13746628; doi:10.1186/s12882-026-05240-1)
Supplement: Supplementary file 1 [file 12882_2026_5240_MOESM1_ESM.pdf]

**Additional file 4: Clinician interview topic guide: Pilot Intervention**

Optimising Staff-patient Communication in Advanced Renal disease

(The **OSCAR** study)

**Clinician interview topic guide: Pilot Intervention**

**A. Overall**

Now that some time has elapsed, please could you share your reflections on the training?

**B. Acceptability and feasibility of each component of the intervention**

How did you find the training being delivered online? *Chat function?*

What are your thoughts on training as a multidisciplinary team? *(i.e. not only with other doctors/nurses)*

What are your views on the overall length of the training?

What are your views on the use of real consultation clips?

**C. Impact on practice**

What are your key takeaway messages from the training?

Please can you tell me about any skills or knowledge that you learned in the pilot communication training intervention that you have since used?

Thinking about the conversation map, do you have any views on this? *Potential prompts: Is it useful clinically? Do you see value in the framework presented? Note: may need to remind the participant of what the map covered here.*

In what way, if any, has the training changed your approach to discussing treatment options with patients who have advanced kidney disease?

Do you think the training will impact or has already impacted outcomes for patients?

**D. Barriers to and facilitators of its implementation**

Please can you tell me about any barriers that you envisage or have experienced, to the implementation of the skills or knowledge that were shared in the intervention?

Please can you tell me about any potential facilitators that you have identified, for implementing the skills or knowledge that were shared in the intervention? *If prompting required: have you found any ways it's been made easy to implement the things you have learned?*

**E. Further comments:**

Thank you for your time. Do you have any other questions or comments? Is there anything I did not ask about that might be useful for us to know?
