# Supplementary material for: ‘Talk CKD Options’: improving how clinicians communicate treatment options in advanced kidney disease using a conversation analysis-based training intervention
Source: BMC Nephrol. 2026 Aug 11;27:656. doi: 10.1186/s12882-026-05240-1 (PMC13746628; doi:10.1186/s12882-026-05240-1)
Supplement: Supplementary file 2 [file 12882_2026_5240_MOESM2_ESM.pdf]

Additional file 1: Learning Objectives

1. To summarise the existing observational evidence about dialysis outcomes in the elderly and/or multimorbid population
2. To understand how older people make the decision about treatment options in advanced kidney disease
3. To identify how the way we work and the environment we work in can impede our ability to support people
4. To develop a conversation map and toolkit to provide person-centred support for treatment decision making in advanced kidney disease
