# Supplementary material for: ‘Talk CKD Options’: improving how clinicians communicate treatment options in advanced kidney disease using a conversation analysis-based training intervention
Source: BMC Nephrol. 2026 Aug 11;27:656. doi: 10.1186/s12882-026-05240-1 (PMC13746628; doi:10.1186/s12882-026-05240-1)
Supplement: Supplementary file 3 [file 12882_2026_5240_MOESM3_ESM.pdf]

# Pre-Training Survey

This survey explores previous training you may have received about communicating with patients regarding treatment options for advanced kidney disease. It also asks you to self-report your knowledge, ability and confidence in this topic.
This survey should be completed prior to starting the Talk CKD Options training.

## About you

1. Job Title *

0/32,000 characters

2. Grade *

0/32,000 characters

3. Specific role in CKD care *

0/32,000 characters

4. Years in this role *

0/32,000 characters

5. Hospital *

0/32,000 characters

## Previous Training

6. Up to now, have you had any formal training about communicating with patients about treatment options for advanced kidney disease? *

◯ Yes
◯ No

7. If yes, was this: (please select all applicable)

☐ In-person
☐ Online, facilitated
☐ Online, self-guided/no facilitator

8. How long ago was the most recent training you completed in this area?

◯ 0-6 months ago
◯ 6-12 months ago
◯ 1-3 years ago
◯ 3-5 years ago
◯ > 5 years ago

9. Was the most recent training you completed:

◯ During specialist training
◯ Continuing professional development (CPD)
◯ Other (include further details below)

10. Please tell us more about the training you have attended in your own words (e.g. duration, content, format, views on effectiveness)

0/32,000 characters

## Knowledge in communicating with patients about treatment options for advanced kidney disease (including conservative kidney management)

Please select the number which represents your level of knowledge for each of the following statements, on a scale of 1-5, with 1 being the lowest and 5 being the highest value.

11. My level of **knowledge** in communicating with patients about treatment options for advanced kidney disease is: *

- ○ 1 (Not knowledgeable at all)
- ○ 2
- ○ 3
- ○ 4
- ○ 5 (Very knowledgeable)

12. To what extent do you know about... *

The survival outcomes for the treatment options available to patients with advanced kidney disease?

- ○ 1 (Not knowledgeable at all)
- ○ 2
- ○ 3
- ○ 4
- ○ 5 (Very knowledgeable)

The quality of life outcomes for the treatment options available to patients with advanced kidney disease?

- ○ 1 (Not knowledgeable at all)
- ○ 2
- ○ 3
- ○ 4
- ○ 5 (Very knowledgeable)

The advantages and disadvantages of the treatment options available to patients with advanced kidney disease?

- ○ 1 (Not knowledgeable at all)
- ○ 2
- ○ 3
- ○ 4
- ○ 5 (Very knowledgeable)

What factors are important to people when making treatment decisions for advanced kidney disease?

- ○ 1 (Not knowledgeable at all)
- ○ 2
- ○ 3
- ○ 4
- ○ 5 (Very knowledgeable)

## Ability in communicating with patients about treatment options for advanced kidney disease (including conservative kidney management)

Please select the number which represents your view for each of the following statments, on a scale of 1-5, with 1 being the lowest and 5 being the highest value.

13. My level of **skill** in communicating with patients about treatment options for advanced kidney disease *

- ◯ 1 (Not skilled at all)
- ◯ 2
- ◯ 3
- ◯ 4
- ◯ 5 (Very skilled)
- ◯ Don't know/not sure/not applicable

14. To what extent do you currently feel **equipped** to communicate with patients about treatment options for advanced kidney disease in a way that... *

Is balanced?

- ◯ Not at all
- ◯ Very little
- ◯ Somewhat
- ◯ To a reasonable extent
- ◯ To a great extent

Helps patients engage with all treatment options?

- ◯ Not at all
- ◯ Very little
- ◯ Somewhat
- ◯ To a reasonable extent
- ◯ To a great extent

Enables patients to ask questions and express their perspectives about all treatment options?

- ◯ Not at all
- ◯ Very little
- ◯ Somewhat
- ◯ To a reasonable extent
- ◯ To a great extent

## Confidence in communicating with patients about treatment options for advanced kidney disease (including conservative kidney management)

Please select the number which represents your view for each of the following statments, on a scale of 1-5, with 1 being the lowest and 5 the highest value.

15. My level of **confidence** in communicating with patients about treatment options for advanced kidney disease is *

- ◯ 1 (Not confident at all)
- ◯ 2
- ◯ 3
- ◯ 4
- ◯ 5 (Very confident)

16. To what extent are you **confident** in communicating with patients about tretament options for advanced kidney disease, in a way that: *

Is balanced?

- ◯ 1 (Not confident at all)
- ◯ 2
- ◯ 3
- ◯ 4
- ◯ 5 (Very confident)

Helps patients engage with all treatment options?

- ◯ 1 (Not confident at all)
- ◯ 2
- ◯ 3
- ◯ 4
- ◯ 5 (Very confident)

Enables patients to ask questions and express their perspectives about all treatment options?

- ◯ 1 (Not confident at all)
- ◯ 2
- ◯ 3
- ◯ 4
- ◯ 5 (Very confident)

## Demographic details

17. Gender

- ◯ Woman
- ◯ Man
- ◯ Non-binary
- ◯ Prefer not to say
- ◯ Prefer to self describe

18. Prefer to self describe gender:

0/32,000 characters

19. Age in years

0/32,000 characters

20. Ethnicity

- ◯ White
- ◯ Mixed / Multiple ethnic groups
- ◯ Asian / Asian British
- ◯ Black / African / Caribbean / Black British
- ◯ Other ethnic group

21. White ethnicity background

- ◯ English / Welsh / Scottish / Northern Irish / British
- ◯ Irish
- ◯ Gypsy or (Irish) Traveller
- ◯ Other

22. Mixed / multiple ethnic background

- ◯ White and Black Carribean
- ◯ White and Black African
- ◯ White and Asian
- ◯ Any other mixed / multiple ethnic background

23. Asian / Asian British background

- ◯ Indian
- ◯ Pakistani
- ◯ Bangladeshi
- ◯ Chinese
- ◯ Any other Asian / Asian British background

24. Black / African / Carribean / Black British background

- ◯ African
- ◯ Caribbean
- ◯ Any other Black / African / Caribbean background

25. Other ethnic group

- ◯ Arab
- ◯ Any other background
