# Supplementary material for: ‘Talk CKD Options’: improving how clinicians communicate treatment options in advanced kidney disease using a conversation analysis-based training intervention
Source: BMC Nephrol. 2026 Aug 11;27:656. doi: 10.1186/s12882-026-05240-1 (PMC13746628; doi:10.1186/s12882-026-05240-1)
Supplement: Supplementary file 4 [file 12882_2026_5240_MOESM4_ESM.pdf]

Post-Training Survey

Thank you for taking the time to complete this post-training survey, following your attendance at the Talk CKD Options pilot training.

Learning Objectives

1. Were the learning objectives of the training met? (Learning objectives listed in table below) *

Summarise the existing observational evidence about dialysis outcomes in the elderly and/or multimorbid population

- ◯ Not at all
- ◯ To a very minimal extent
- ◯ Somewhat
- ◯ To a reasonable extent
- ◯ To a great extent

Understand how older people make the decision about treatment options in advanced kidney disease

- ◯ Not at all
- ◯ To a very minimal extent
- ◯ Somewhat
- ◯ To a reasonable extent
- ◯ To a great extent

Identify how the way we work and the environment we work in can impede our ability to support people

- ◯ Not at all
- ◯ To a very minimal extent
- ◯ Somewhat
- ◯ To a reasonable extent
- ◯ To a great extent

Develop a conversation map and toolkit to provide person-centred support for treatment decision making in advanced kidney disease

- ◯ Not at all
- ◯ To a very minimal extent
- ◯ Somewhat
- ◯ To a reasonable extent
- ◯ To a great extent

Your current knowledge in communicating with patients about treatment options for advanced kidney disease (including conservative kidney management)

Please select the number which represents your view for each of the following statements, on a scale of 1-5, with 1 being the lowest and 5 the highest value.

2. My level of **knowledge** in communicating with patients about treatment options for advanced kidney disease is now: *

- ◯ 1 (Not knowledgeable at all)
- ◯ 2
- ◯ 3
- ◯ 4
- ◯ 5 (Very knowledgeable)

3. To what extent do you know about... *

The survival outcomes for the treatment options available to patients with advanced kidney disease?

- ◯ 1 (Not knowledgeable at all)
- ◯ 2
- ◯ 3
- ◯ 4
- ◯ 5 (Very knowledgeable)

The quality of life outcomes for the treatment options available to patients with advanced kidney disease?

- ◯ 1 (Not knowledgeable at all)
- ◯ 2
- ◯ 3
- ◯ 4
- ◯ 5 (Very knowledgeable)

The advantages and disadvantages of the treatment options available to patients with advanced kidney disease?

- ◯ 1 (Not knowledgeable at all)
- ◯ 2
- ◯ 3
- ◯ 4
- ◯ 5 (Very knowledgeable)

What factors are important to people when making treatment decisions for advanced kidney disease?

- ◯ 1 (Not knowledgeable at all)
- ◯ 2
- ◯ 3
- ◯ 4
- ◯ 5 (Very knowledgeable)

Ability in communicating with patients about treatment options for advanced kidney disease (including conservative kidney management)

Please select the number which represents your view for each of the following statements, on a scale of 1-5, with 1 being the lowest and 5 the highest value.

4. My level of **skill** in communicating with patients about treatment options for advanced kidney disease is now: *

- ◯ 1 (Not skilled at all)
- ◯ 2
- ◯ 3
- ◯ 4
- ◯ 5 (Very skilled)

5. To what extent do you currently feel **equipped** to communicate with patients about treatment options for advanced kidney disease in a way that... *

Is balanced?

- ◯ 1 (Not at all)
- ◯ 2
- ◯ 3
- ◯ 4
- ◯ 5 (To a great extent)

Helps patients engage with all treatment options?

- ◯ 1 (Not at all)
- ◯ 2
- ◯ 3
- ◯ 4
- ◯ 5 (To a great extent)

Enables patients to ask questions and express their perspectives about all treatment options?

- ◯ 1 (Not at all)
- ◯ 2
- ◯ 3
- ◯ 4
- ◯ 5 (To a great extent)

Confidence in communicating with patients about treatment options for advanced kidney disease (including conservative kidney management)

Please select the number which represents your view for each of the following statements, on a scale of 1-5, with 1 being the lowest and 5 the highest value.

6. My level of **confidence** in communicating with patients about treatment options for advanced kidney disease is now: *

- ◯ 1 (Not confident at all)
- ◯ 2
- ◯ 3
- ◯ 4
- ◯ 5 (Very confident)

7. To what extent are you **confident** in communicating with patients about tretament options for advanced kidney disease, in a way that... *

Is balanced?

- ◯ 1 (Not confident at all)
- ◯ 2
- ◯ 3
- ◯ 4
- ◯ 5 (Very confident)

Helps patients engage with all treatment options?

- ◯ 1 (Not confident at all)
- ◯ 2
- ◯ 3
- ◯ 4
- ◯ 5 (Very confident)

Enables patients to ask questions and express their perspectives about all treatment options?

- ◯ 1 (Not confident at all)
- ◯ 2
- ◯ 3
- ◯ 4
- ◯ 5 (Very confident)

Pre-training content

This section relates to the pre-recorded, pre-training content you were invited to view.,

8. I watched both pre-recorded presentations in advance of attending the training. *

- ◯ Yes
- ◯ No

9. Please give the main reason why you were unable to watch the pre-recorded presentations

0/32,000 characters

10. How would you rate each presentation?

Fliss Murtagh: Current evidence on outcomes in advanced kidney disease

- ◯ Very Poor
- ◯ Poor
- ◯ Fair
- ◯ Good
- ◯ Very Good

Barny Hole: Values, goals and uncertain futures

- ◯ Very Poor
- ◯ Poor
- ◯ Fair
- ◯ Good
- ◯ Very Good

11. Please expand on your rating for Fliss Murtagh's pre-recorded presentation.

0/32,000 characters

12. Please expland on your rating for Barny Hole's pre-recorded presentation.

0/32,000 characters

The Training Format and Structure

13. To what extent do you think the overall mode of the training (being online, live and interactive) worked well? *

- ◯ Not at all
- ◯ To a very minimal extent
- ◯ Somewhat
- ◯ To a reasonable extent
- ◯ To a great extent

14. If the training was online, but you could access it in your own time (rather than being live and interactive), to what extent do you think that would work well? *

- ◯ Not at all
- ◯ To a very minimal extent
- ◯ Somewhat
- ◯ To a reasonable extent
- ◯ To a great extent

15. To what extent do you think training as a multidisciplinary team worked well? (as opposed to only training with other doctors/nurses etc) *

- ◯ Not at all
- ◯ To a very minimal extent
- ◯ Somewhat
- ◯ To a reasonable extent
- ◯ To a great extent

16. The overall length of the live training session was... *

- ◯ Far too short
- ◯ Slightly short
- ◯ A good length
- ◯ Slightly long
- ◯ Far too long

17. The number of consultation video examples used was... *

- Far too few
- Slightly too few
- A good amount
- Slightly too many
- Far too many

18. Were there any parts of the training which you felt needed shortening? *

- Yes
- No

19. Please expand on which parts you felt needed shortening:

0/32,000 characters

20. Were there any parts of the training which you felt needed more time spent on them? *

- Yes
- No

21. Please expand on which parts you felt needed more time spent on them:

0/32,000 characters

22. During the live training, I understood the point we had reached in the training and where we were heading next. *

- Strongly disagree
- Disagree
- Agree
- Strongly agree

The Live Training Session

23. How would you rate the breakout group sessions? *

- Very Poor
- Poor
- Fair
- Good
- Very Good

24. Please expand on your rating for the breakout group sessions:

0/32,000 characters

25. The facilitator of my breakout group discussions was... *

- Chloe Shaw
- Lucy Selman
- Barny Hole

26. How would you rate the facilitation of your breakout group sessions? *

- Very Poor
- Poor
- Fair
- Good
- Very Good

27. Please share your views on how well your breakout group sessions were facilitated:

0/32,000 characters

28. How would you rate the sessions delivered to the whole group? *

- ◯ Very Poor
- ◯ Poor
- ◯ Fair
- ◯ Good
- ◯ Very Good

29. Please expand on your rating:

0/32,000 characters

Training Environment

30. What did you think of the quality of the online platform that was used (e.g. usability, connection quality)? *

- ◯ Very Poor
- ◯ Poor
- ◯ Fair
- ◯ Good
- ◯ Very Good

31. What made the quality of the online platform poor or good?

0/32,000 characters

Overall Feedback

32. How likely would you be to recommend the training to a colleague? *

- ◯ Very Unlikely
- ◯ Unlikely
- ◯ Neutral
- ◯ Likely
- ◯ Very Likely

33. To what extent do you think the training will influence your practice? *

- ◯ Not at all
- ◯ To a very minimal extent
- ◯ Somewhat
- ◯ To a reasonable extent
- ◯ To a great extent

34. What is the single most important thing you will take away from the training and apply in your work? *

0/32,000 characters

35. What information in this training surprised, inspired and/or helped you most? *

0/32,000 characters

36. What, if anything, would have improved the training workshop?

0/32,000 characters

37. Was there any bias or conflict of interest evident in the course? *

- ◯ Yes
- ◯ No

38. Please expand on your answer:

0/32,000 characters
