# Supplementary material for: ‘Talk CKD Options’: improving how clinicians communicate treatment options in advanced kidney disease using a conversation analysis-based training intervention
Source: BMC Nephrol. 2026 Aug 11;27:656. doi: 10.1186/s12882-026-05240-1 (PMC13746628; doi:10.1186/s12882-026-05240-1)
Supplement: Supplementary file 6 [file 12882_2026_5240_MOESM6_ESM.pdf]

Additional file 6: Coding framework for qualitative data analysis

Theme	Sub-theme	Code
Acceptability and feasibility	Overall positive	
	Amount of content	Right amount of content
		Too much information
	Breakout groups	Positive about breakout groups
		Well facilitated
		Reluctance to contribute in big groups
	Length	Appropriate length training
		Needed more time
	MDT Learning	Positive about MDT Learning
		Impact of perceived hierarchy
		Clinician bias
	Online delivery	Positive: Access wider cohort online
		Positive: Feasibility trade off
		Teams chat function positive
		Teams chat function distracting
Impact on practice	Takeaway messages	Equitable discussion of options
		Person centred approach
		The importance of language
		The importance of evidence
	Practice changing	Patient voice videos powerful
	Sharing knowledge with others	
	Knowledge	Building on prior knowledge
	Ability	Reinforcing current practice
	Confidence	
	Which staff would benefit	Beneficial across MDT and seniority
		Beneficial to registrars
		Beneficial to new starters
		Difficult for more junior staff
Potential impact on patient outcomes	Would impact outcomes	
	Difficult to measure outcomes	
Barriers to implementation	System Factors	Time as a barrier
		Variation in CKM services offered
	Patient Factors	Impact of pre-conceived ideas about treatments
		Challenging if conflict with family
		Cultural barriers
Limitations of training		Didn't cover cultural differences
		Knowledge retention of conversation map
Suggestions for improvements	Supplemental training materials	Access to slides
		Desire for a post-session handout
	Lengthen training	Divide into two sessions
		More time on communication tools
		More time in breakout groups
